# A Regenerative Breakthrough in the Management of a Cystic Lesion Using a Novel Combination of Eggshell-Derived Nanohydroxyapatite (EnHA) and Concentrated Growth Factor: A Case Report

**DOI:** 10.7759/cureus.113568

**Published:** 2026-07-29

**Authors:** Ansh Gujral, Vikrant Sane, Adhishree S Joshi, Vivek S Nair, Rohit Thorat, Rashmi Sane

**Affiliations:** 1 Oral and Maxillofacial Surgery, Bharati Vidyapeeth (Deemed to be University) Dental College and Hospital, Pune, IND; 2 Prosthodontics, Bharati Vidyapeeth (Deemed to be University) Dental College and Hospital, Pune, IND; 3 Oral Medicine and Radiology, Bharati Vidyapeeth (Deemed to be University) Dental College and Hospital, Pune, IND

**Keywords:** concentrated growth factor, cyst, cystic defect, grafting, nanohydroxyapatite

## Abstract

Eggshell-derived nanohydroxyapatite (EnHA) is an economical, bioactive, and biocompatible bone graft substitute that has been shown to promote active bone formation. It has previously been used in socket preservation and periodontal defects. Autologous blood products such as platelet-rich plasma (PRP), platelet-rich fibrin (PRF), and concentrated growth factor (CGF) are being studied extensively for their role in bone regeneration. CGF has shown osteoinductive properties by promoting vascularization and early angiogenesis. Surgical enucleation of a cystic lesion measuring 17.7 × 15.1 mm was performed, followed by grafting of the bony defect using EnHA combined with autologous CGF. Through this case report, we aim to evaluate the feasibility and preliminary outcome of EnHA combined with CGF for bone regeneration in an intrabony defect after removal of a cystic lesion in the posterior mandible.

## Introduction

Dental cyst enucleation often leads to a bony defect requiring regenerative intervention. Various bone graft substitutes have been used to promote the healing of intrabony defects in maxillofacial surgery. Although autogenous bone grafts are regarded as the gold standard, their use is constrained by donor-site morbidity, increased surgical time, and restricted availability. Alternative graft materials such as allografts, xenografts, and alloplasts have been successfully used. However, each presents inherent limitations related to cost, availability, and biological performance. Eggshell-derived hydroxyapatite has recently emerged as a viable biomaterial with physicochemical characteristics closely resembling those of natural bone mineral. CGF is an autologous platelet aggregate with wide applications in maxillofacial surgery and good regenerative potential. It is enriched with platelets, leukocytes, growth factors, stem cells, and fibrin [1]. Individually, both EnHA and CGF have been used in socket preservation, periodontal defects, direct sinus augmentation, and other areas where bone regeneration is required. However, evidence regarding their combined use is limited. Based on PubMed and Scopus searches using the keywords “eggshell-derived hydroxyapatite” and “concentrated growth factor,” limited prior studies have been reported on the use of this combination [2,3].

Nanoparticles in EnHA provide a biocompatible scaffold that mimics bone. The addition of CGF enhances healing through the steady and gradual release of autologous growth factors [4].

In this case report, the combination was used for an intrabony defect created in the posterior mandibular region after enucleation of a large radicular cyst.

## Case presentation

**Figure 1:**
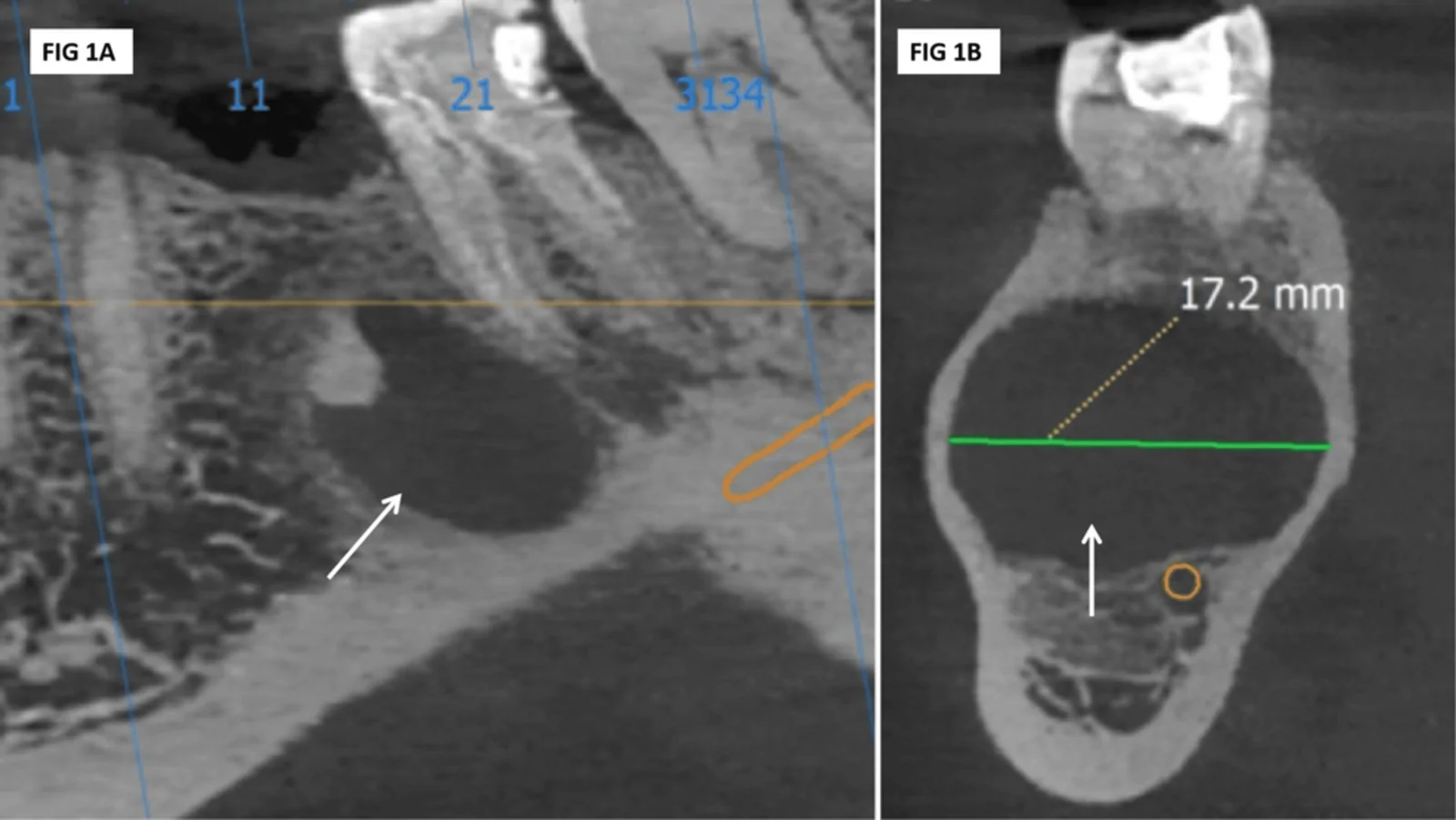
Pre-operative radiographic analysis by cone beam computed tomography (CBCT) (A) The arrow shows radiolucency of the cystic lesion in panoramic view. (B) The arrow shows radiolucency of the cystic lesion in the sagittal view.

**Figure 2:**
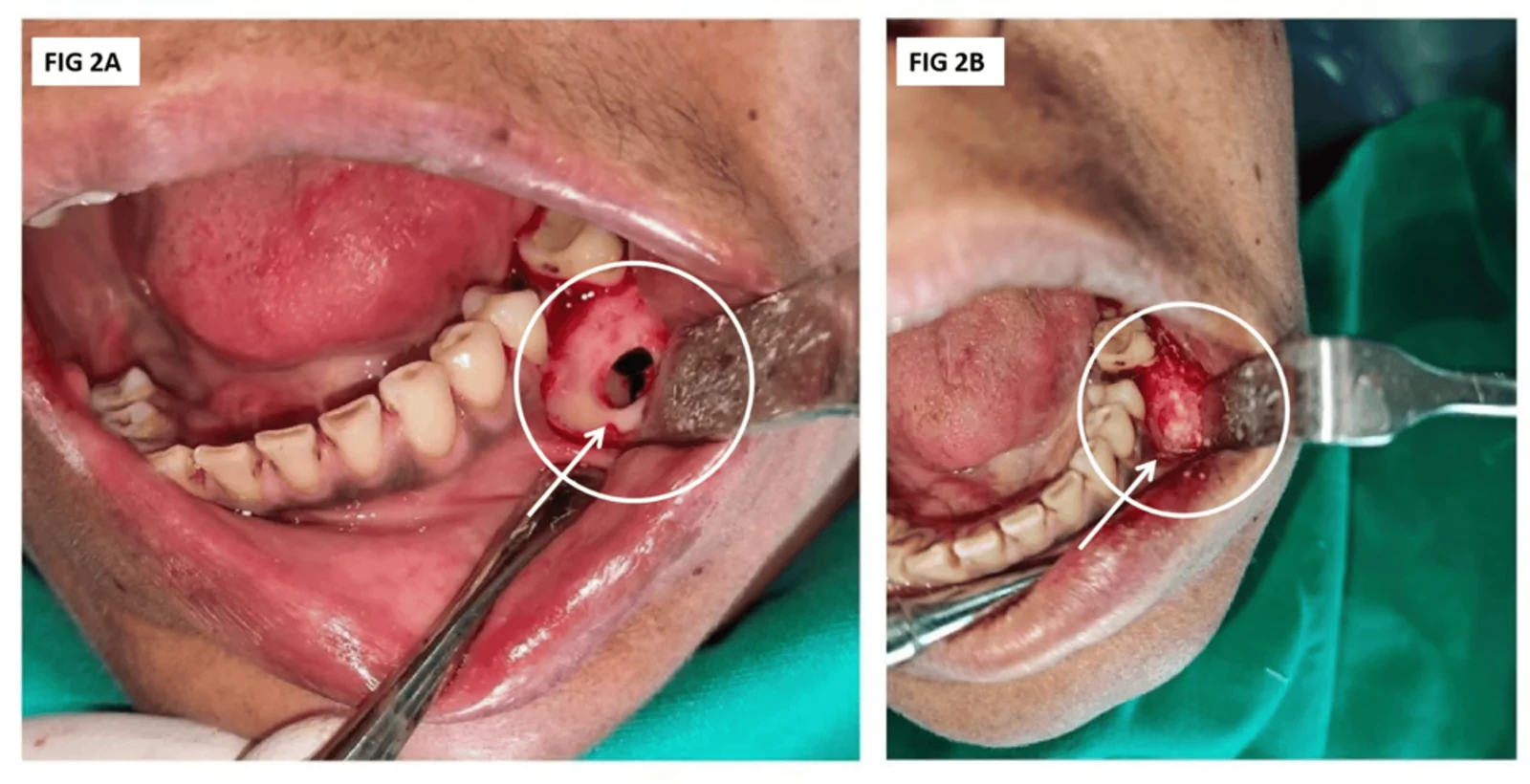
Intraoperative images (A) The arrow shows a bony window created for the enucleation of a cystic lesion. (B) The arrow shows grafting of the cystic defect with eggshell-derived nanohydroxyapatite mixed with concentrated growth factor.

The patient presented to the Department of Oral and Maxillofacial Surgery with a chief complaint of pain and swelling in the lower left posterior region of the jaw. Cone-beam computed tomography was advised, which revealed a cystic lesion measuring 17.7 × 15.1 mm associated with an odontome in the left mandibular first molar region (Figures 1A, 1B). After hematological assessment, the patient underwent surgical enucleation of the cyst. A crevicular incision extending from 34 to 37 was made, with a releasing incision on the mesial side of 34. A bony cavity was created using the postage stamp technique (Figure 2A). The cyst was enucleated along with the odontome, followed by grafting of the bony defect using indigenously prepared EnHA bone graft combined with autologous CGF (Figure 2B). Primary closure was performed using 3-0 Mersilk (Ethicon, Inc., Raritan, New Jersey).

Procedure for preparation of the EnHA graft [5]

Eggshell-derived hydroxyapatite (EnHA) was produced and procured from NHAP Regenerative Solutions Pvt. Ltd. The production methodology involved sequential steps: eggshells were cleaned, dried, powdered, treated with sodium hypochlorite (NaOCl), thoroughly washed, vacuum-dried, converted into a calcium-EDTA complex, and subsequently reacted with disodium hydrogen orthophosphate under alkaline conditions with microwave irradiation to yield hydroxyapatite. The resulting HA particles measured 0.5-1 µm in length and 100-200 nm in width.

The EnHA fulfilled the essential criteria of an ideal graft substitute, as its nanoscale particle size and porous structure, combined with calcium deficiency, closely resembled the properties of native bone hydroxyapatite. The material was sterilized using gamma radiation, and preclinical toxicology testing was performed in accordance with ISO 10993 standards for cytotoxicity, sterility, and biocompatibility in a Central Drugs Standard Control Organization (CDSCO)-approved laboratory.

Procedure for making the CGF plug

Venous blood (10 mL) was collected from the patient's antecubital fossa. The sample was promptly centrifuged at variable speeds using a preprogrammed centrifuge system (30-second acceleration, 2700 rpm for four minutes, 3000 rpm for three minutes, and 36-second deceleration) (T-ENSHIN) to obtain concentrated growth factor (CGF) plugs. The CGF plugs were subsequently mixed with eggshell-derived nanohydroxyapatite (EnHA) graft material and utilized for sinus augmentation.

Radiologic analysis

**Figure 3:**
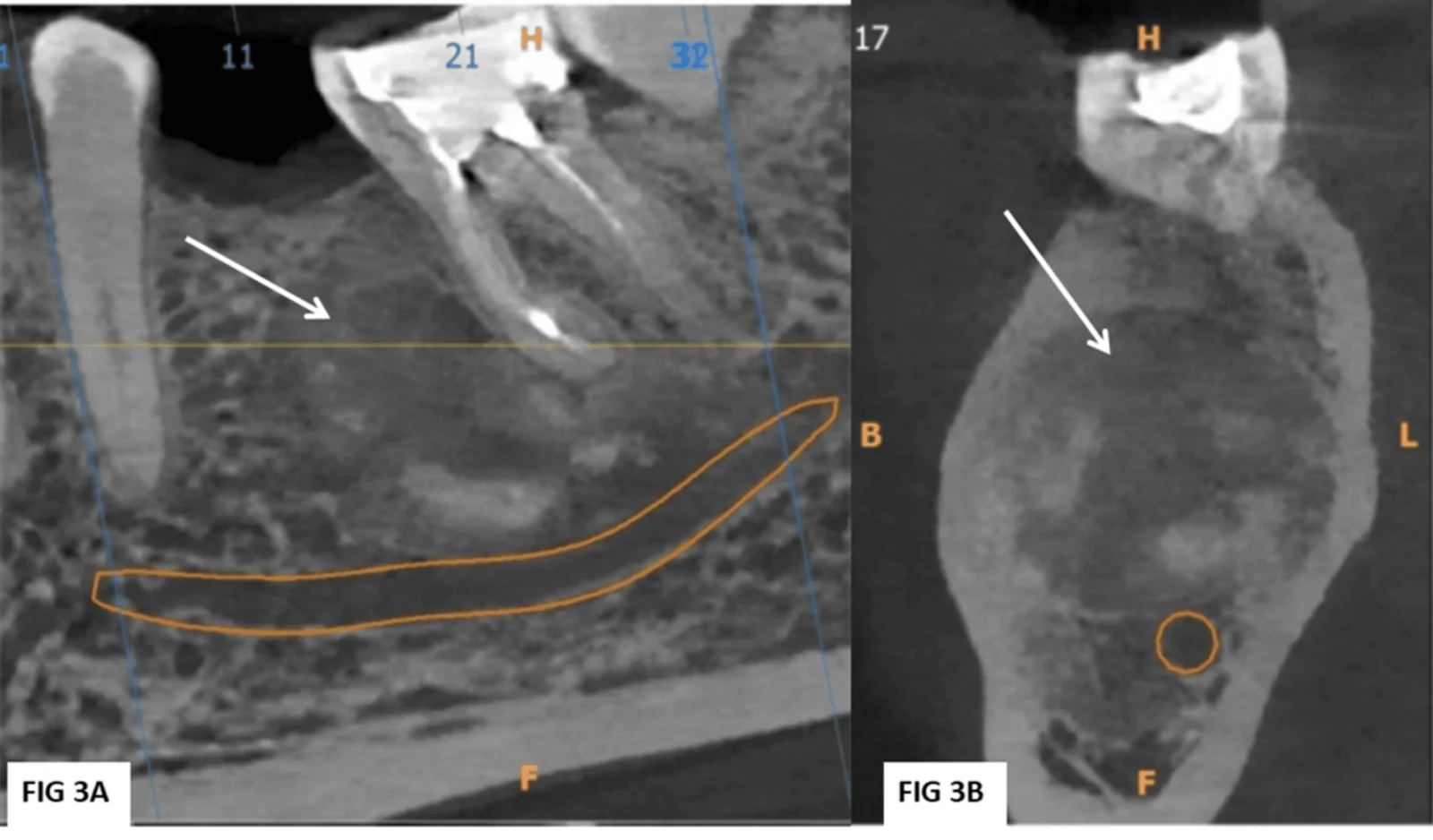
Cone beam computed tomography (CBCT) images at six-month follow-up (A) The arrow indicates the grafted site, which shows radio-opacity and new bone formation in the panoramic view. (B) The arrow indicates the grafted site, which shows radio-opacity and new bone formation in the sagittal view.

Patients were recalled for follow-up after three and six months. Postoperative cone beam computed tomography (CBCT) obtained after 6 months demonstrated satisfactory bone regeneration within the grafted site, with bone density corresponding to Type 3 bone quality according to the Lekholm and Zarb bone classification, indicating satisfactory bone formation at the grafted site [6] (Figures 3A, 3B).

Histopathological analysis

When the site was revisited for dental implant placement after six months, a trephine was used to retrieve a piece of bony tissue for histopathological examination (HPE). The specimen was fixed in 10% formalin and decalcified using 5% nitric acid for 36-48 hours. The samples were then processed, and paraffin-embedded blocks were prepared. Sections measuring 3-4 µm were prepared using a rotary microtome, followed by hematoxylin and eosin staining. The slides were observed under a trinocular research microscope at 10× magnification.

**Figure 4:**
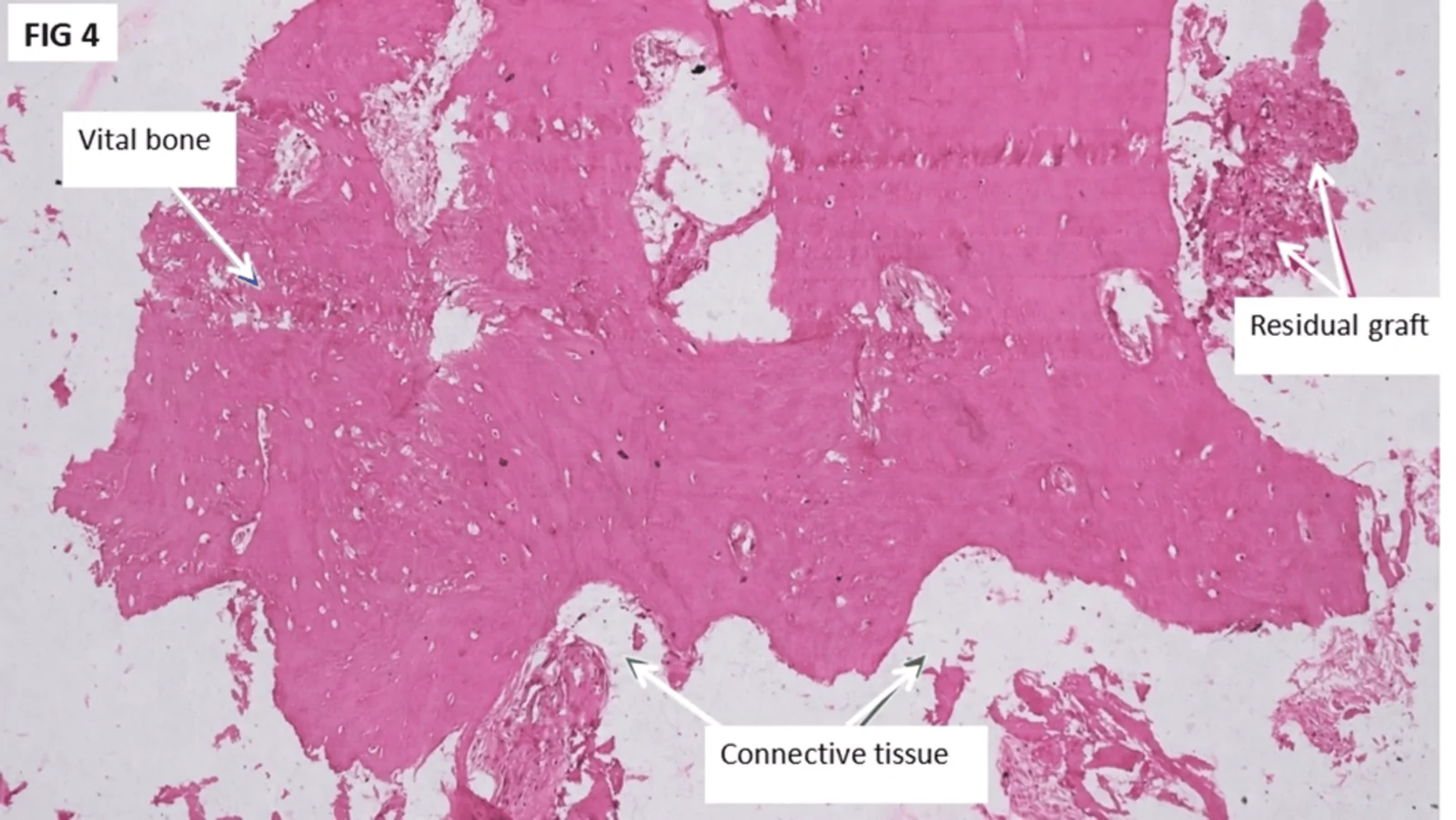
Hematoxylin and eosin staining of bony specimen under 10X magnification The arrows show vital bone, connective tissue, and residual graft material.

HPE revealed newly formed bone and connective tissue with blood elements. Areas of bone marrow and some residual graft material were also seen. The bone tissue consisted of trabeculae with embedded osteocytes, suggestive of active bone formation. No necrosis or inflammatory cells were observed. These findings confirmed active bone formation and a favorable tissue response within the grafted site (Figure 4).

Histomorphometric analysis

Quantitative analysis was performed to evaluate the percentage distributions of vital bone, connective tissue and marrow space, and residual graft material using JENOPTIK GRYPHAX image analysis software (JENOPTIK AG, Jena, Germany). The histological sections were observed under a trinocular research microscope connected to a high-resolution digital camera and a computer-based imaging system. The microscopic images captured were analyzed using calibrated tools. The measurements obtained in square micrometers were converted into percentage values relative to the total tissue area analyzed. The values obtained are presented in Table 1.

**Table 1:** Quantitative analysis of bony specimen using JENOPTIK GRYPHAX image analysis software (JENOPTIK AG, Jena, Germany)

Variables	Values
Vital bone	98.03%
Connective tissue	1.00%
Residual graft material	0.97%

## Discussion

EnHA has recently emerged as an economical and biocompatible bone graft substitute in oral and maxillofacial surgery. Several studies have demonstrated its osteoconductive potential in the management of cystic defects, extraction sockets, and periodontal intrabony defects.

Kattimani et al. reported significant bone regeneration in maxillary cystic defects grafted with EnHA, with radiographic evidence of trabecular bone formation and satisfactory healing outcomes [7]. Further randomized clinical studies also confirmed that EnHA enhances bone density and promotes early osseous healing compared with conventional hydroxyapatite grafts.

Ganapathy et al. reported the potential of EnHA as a bone graft substitute in intrabony defects based on both clinical and CBCT evaluations during a six-month observation period [8].

The incorporation of CGF or platelet concentrates with hydroxyapatite-based grafts has shown additional regenerative benefits because CGF provides a rich source of growth factors that accelerate angiogenesis, osteoblastic activity, and soft tissue healing [9]. Studies using platelet-rich fibrin (PRF) and injectable PRF (i-PRF) in combination with hydroxyapatite demonstrated improved socket preservation and faster bone maturation in oral surgical defects. Kattimani et al. also described socket preservation using EnHA combined with PRF as a barrier membrane, reporting satisfactory bone fill and uneventful healing [4].

Although the literature specifically evaluating EnHA combined with CGF is still limited, existing evidence suggests that combining eggshell-derived hydroxyapatite with autologous growth factor concentrates can provide synergistic effects on bone regeneration. This combination appears to be a cost-effective, eco-friendly alternative to other available synthetic graft materials because it utilizes a readily available biomaterial, thus reducing the need for additional processing and expense. It is osteoconductive due to its nanoparticle size. Mixing it with autologous CGF gives it osteoinductive and osteogenic potential, making it a desirable alternative to synthetic graft materials for bone regeneration procedures. Growth factors in CGF, such as vascular endothelial growth factor (VEGF), platelet-derived growth factor (PDGF), and transforming growth factor beta (TGF-β), promote early vascularization (blood vessel formation) and cell proliferation, which accelerate the overall bone formation and maturation process [10].

## Conclusions

Preliminary observations from our case report suggest that EnHA mixed with CGF has potential for bone regeneration in cystic defects. It is a viable and economical choice compared with other graft materials. It is easy to prepare, and the raw material is available in plenty. In a developing country like India, where synthetic bone grafts are not affordable for everyone, EnHA can be a real game-changer. However, comparative studies, larger clinical trials, and longer follow-up would be beneficial to study the long-term behavior of this bone graft material in combination with CGF.
